# Hybrid fuzzy deep neural network toward temporal-spatial-frequency features learning of motor imagery signals

**DOI:** 10.1038/s41598-022-26882-9

**Published:** 2022-12-25

**Authors:** Maryam Sorkhi, Mohammad Reza Jahed-Motlagh, Behrouz Minaei-Bidgoli, Mohammad Reza Daliri

**Affiliations:** 1 School of Computer Engineering, Iran University of Science and Technology, Tehran, Iran; 2 School of Electrical Engineering, Iran University of Science and Technology, Tehran, Iran

**Keywords:** Electrical and electronic engineering, Cognitive neuroscience

## Abstract

Achieving an efficient and reliable method is essential to interpret a user’s brain wave and deliver an accurate response in biomedical signal processing. However, EEG patterns exhibit high variability across time and uncertainty due to noise and it is a significant problem to be addressed in mental task as motor imagery. Therefore, fuzzy components may help to enable a higher tolerance to noisy conditions. With the advent of Deep Learning and its considerable contributions to Artificial intelligence and data analysis, numerous efforts have been made to evaluate and analyze brain signals. In this study, to make use of neural activity phenomena, the feature extraction preprocessing is applied based on Multi-scale filter bank CSP. In the following, the hybrid series architecture named EEG-CLFCNet is proposed which extract the frequency and spatial features by Compact-CNN and the temporal features by the LSTM network. However, the classification results are evaluated by merging the fully connected network and fuzzy neural block. Here, the proposed method is further validated by the BCI competition IV-2a dataset and compare with two hyperparameter tuning methods, Coordinate-descent and Bayesian optimization algorithm. The proposed architecture that used fuzzy neural block and Bayesian optimization as tuning approach, results in better classification accuracy compared with the state-of-the-art literatures. As results shown, the remarkable performance of the proposed model, EEG-CLFCNet, and the general integration of fuzzy units to other classifiers would pave the way for enhanced MI-based BCI systems.

## Introduction

Brain Computer Interface (BCI) https://www.sciencedirect.com/topics/medicine-and-dentistry/brain-computer-interface could assist, strengthen, or recover human cognitive and sensory-motor tasks^1,2^. In BCI systems, to attain the user's brain signal, among non-invasive methods, Electroencephalography (EEG) is widely used because of its great temporal precision, less risk, and simple setup^3^. One of the EEG application is based on the motor imagery (MI) that produced in the sensory-motor cortex zone of the brain in response to imagery processes^4^. Simple MI-BCI classification methods consistent with the characteristics of EEG signals, could be approximately separated into two groups: classification based on temporal-spatial characteristics^5,6^, and classification based on spatial-frequency characteristics^7–9^,. To extract these features from EEG signals in an incremental manner, deep learning has noteworthy achievements compared to straight hand-crafted methods^10^. In addition, among deep learning methods, Deep Neural Networks (DNN) have become more attractive lately due to greater datasets, superior learning algorithms, and advanced computational speed^11^. Based on DNN networks, Long-Short Term Memory (LSTM) Network and Convolutional Neural Network (CNN) models could learn robust temporal and spatial features^12,13^. Although these features are frequently taken out by deep approaches^14–17^, they are infrequently mined through DNN all at once. However, the accuracy of EEG classification can be enhanced by completely extracting these features together. Furthermore, dissimilar extraction orders might cause diverse classification outcomes. In this case, a hybrid neural network containing LSTM and CNN networks has been exploited in some studies to simultaneous excerpt temporal and spatial features^18–24^.

Although the hybrid technique surpasses high-tech models, it has too numerous limits. Some of these deficiencies are: the overfitting problem when the small dataset is used, the frequency features rarely considered in these models and most of deep learning methods on EEG suppose that EEG patterns are noise-free and stationary^25–29^. As overfitting problem, when the number of convolutional parameters exceed, the train time of hybrid models considerably increases. In this case, execution the Compact-CNN for sorting and analysis of the EEG-based BCIs could help^16^. It was employed to create an EEG-network that compresses numerous distinguished feature extraction, whereas instantaneously the number of trainable parameters for fitting is reduced regarding to current methods. In addition, this network could be applied to expedite the spatial and frequency features detecting process at once.

**Figure 1:**
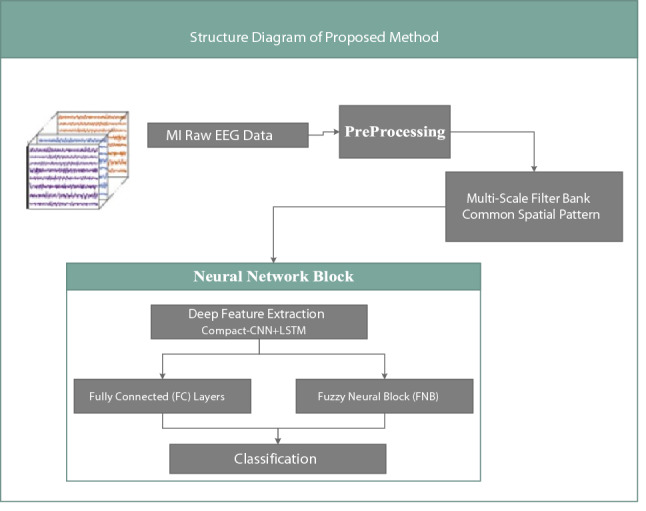
Schematic diagram of the proposed framework comprising.

There are three benefits for the presented idea in this study. Initially, to additional advance in the classification accuracy of EEG signals, the proposed architecture has Multi-scale Filter bank CSP (MSFBCSP)^30^ and Hilbert transform^10^ preprocess for superior filtering, Compact-CNN for extracting the frequency and spatial features of EEG signals, and LSTM for the temporal features detection. In the following, fully connected network (FC) and the fuzzy neural block (FNB) in the last layer are used for classification process. Secondly, proposed model has fewer parameters compare to traditional hybrid models. Associated with conventional CNN, the number of parameters of Compact-CNN is comparatively small. Greater classification accuracy and quicker training speed are the advantages of this method. Lastly, the operation of BCI is enhanced by the reunion of the FNB based on the imagery paradigm in non-optimal environments. The use of FNB in the deep learning architecture was enthused by the fact that the uncertainty of Motor imagery recordings could be increased when more recurrent potential noisier elements and distractors are existing. Subsequently, in this research, it is assumed that the ecological validity of MI-based BCI is improved by accounting the fuzziness in the model pattern parameters and there is theoretically more information confined. Therefore, as displayed in Fig. 1, the above architecture (EEG-CLFCNet) is proposed to mine the temporal-spatial-frequency features concurrently and study the uncertainty of the signals.

The rest of the paper is prepared as follows: Related works are presented in Section “Related work” via reviewing of works on deep learning methods in MI-based BCI. Section “Data”, presents the details of dataset used in this work and Section “Methods”, contains preprocessing and processing approaches, and the whole architecture of the proposed model. In Section “Results and analysis”, the experimental results are presented and the proposed method is compared with preceding investigations. Finally, in Section “Conclusion”, the conclusion of the study is presented.

## Related work

Diverse deep learning and conventional machine learning approaches that have been performed in the BCI rely on MI grouping are reviewed in this section.

To resolve the problem of low signal-to-noise ratio (SNR) in EEG signals, Common Spatial Pattern (CSP) is developed as one of the initial machine learning methods. The goal of this technique is to maximize the discrepancy of one class whereas the variance of other classes is minimized instantaneously^31^. The efficiency of CSP is associated with the subject-specific frequency band^32^. Dissimilar variants of CSP have been suggested to resolve the problem of choosing the optimal frequency band, and between them, Filter Bank Common Spatial Pattern (FBCSP)^9^ through spontaneously choosing subject-specific frequency bands, outperformed other methodologies. Nonetheless, the temporal properties of EEG signals are ignored in all these methods. The classification of EEG signals is done efficiently via CNN due to the ability to learn robust spatial features^1^. The sequential connection of the EEG signal could be efficiently learned by RNN^33^. For unsupervised feature learning, Autoencoder (AE) models are also appropriate^34^. Shallow CNN (sCNN) and Deep CNN (dCNN) architecture are designed by Schirrmeister et al. to learn the temporal assembly of band power variations and to extract a broad variety of features respectively^1^. Nevertheless, because of the restricted number of training data, the dCNN made inferior than the sCNN. Previous studies have indicated that diverse layers of the CNN network could extract diverse concepts of the EEG signals. Thus, extracted features from CNN models are fused by Amin et al. with a numerous number of layers and filters to learn the local and global assembly of time-series signals^35^. The pragmatic CNN (pCNN) network was introduced by Tayeb et al., which is less multifaceted than dCNN and its level of accuracy is high^10^. Besides, the extracted features by CNN are hardly interpreted, so the EEGNet and EEGNet-Inception hybrid neural networks are used by the researchers^15,16^. In the inception network, EEGNet is modified with a superior number of feature maps and the complexity of the model is increased and consequently, this out-performed sCNN. For the arrangement of sequential-spatial-frequency features, Lu et al. employed the deep RNN architecture^36^. In this attitude, individual variances in classification are reduced by considering the signal as the sequences of nonlinear and non-stationary structures. Ma et al. joined the LSTM with bidirectional LSTM (BiLSTM) and examined their method in the eegmmidb dataset to extract spatial and temporal properties^37^. A combination of different neural networks (hybrid DNN) has been extensively employed in BCI to mine spatial and temporal information at once. The Inception architecture is used by Qiao et al. to extract spatial features and then extract temporal features is applied for attaining the Bidirectional Gated Recurrent Unit (BGRU)^38^. Proceeding works revealed that the usage of temporal and spatial features for the classification rises sensitivity to intra-class and inter-class alterations and eventually surges oversimplification. A combination of CNN and LSTM is used to extract frequency-spatial evidence and sequence relation of the signal respectively^19^. They presented that hybrid neural networks executed more efficiently than other neural networks since a wide variety of features are learned simultaneously. Regardless of the success of deep learning, extractions of spatial, temporal and frequency features are rarely done simultaneously and the fuzzy approach is not applicable in these hybrid methods. Lately, Sorkhi et al. applied MSFBCSP, CNN, and tune hyperparameters with Bayesian optimization in their work^20^.

Several scientists have tried to advance the enactment of DNNs in limited-size datasets. The K-fold cross-validation (K-fold CV) is the most conventional technique for this type of dataset. Wang et al. inspected the overall and subject-specific authentication technique^39^. In the overall authentication, the pooled data of some subjects were applied as training data and the remaining subject data was employed as testing data. The cropping technique is proposed by Schirrmeister et al. in which a window slides inside each trial and results in numerous labeled training samples^1^. They found that the performance of the CNN network enhanced when the number of labeled examples is increased. Many other studies applied the same idea to escape overfitting issues and advance network enactment. Furthermore, Lou et al. used the cropping technique to enlarge data for the LSTM model^33^. Lu et al. collected data by using a 2 s sliding window and used them as input for a hybrid CNN and LSTM model^36^.

To extend the time–frequency representation of the data, the deep convolutional Generating Adversarial Network was used by Zhang et al.^21^. In previous research studies, transfer learning has been applied in a variety of arrangements^22,23^. An identical methodology was used to resolve the issue of discrete dissimilarities in the EEG by fine-tuning the internal factors of just the entirely linked layer network for the test data of a new subject^19^. The amount of EEG data is comparatively small due to limited experiments. When P300 evoked abilities are recognized by CNN, Batch Normalization is used to improve overfitting in the input and convolutional layers^40^. Depthwise and separable convolutions are generated as EEGNet, and limited training data with Compact-CNN is used for extraction of the spatial and frequency features^16^. In 2020, parallel and series arrangements combining compact and shallow CNN and LSTM, called SCCRNN, SSCRNN, PCCRNN, and PSCRNN were proposed Wang et al. extracted temporal, frequency, and spatial features and categorize them using these architectures^24^. The regular kappa value of the series configuration with compact CNN for BCI Competition IV 2a Dataset was 0.64, which attained favorable outcomes compared with the other three approaches.

Several researchers have focused on the recognition abilities of regular deep architectures to attain time-course patterns. A novel temporal CNN is signified in the context of Temporal CNN (TCNN) whereas it has exceeded CNN with classical recurrent layers in pattern recognition in signal processing^25^. In addition, a recent trend is an addition of fuzzy logic components into CNN features^26,27^. The key drawback of learning EEG patterns with deep learning is the assumption that EEG patterns are noise-free and not affected by non-stationary, consequently the uncertainty is disregarded. By the way, significant gains are attained by fuzzy sets and systems in noisy BCI problems^28^. Brain-computer interface (BCI) based on P300, using fuzzy temporal convolutional neural networks was proposed by Vega et al. for smart home interactivity^25^. In this study, both stroke survivors and healthy people employed a BCI-enabled smart home.

## Data

In this study, the BCI competition IV-2a data set is used to examine and evaluate our modified algorithm, which is a four-class^41^. This dataset consists of four Motor Imagery(MI) EEG signals of left, right, feet, and tongue recorded from 22 electrodes with a 250-Hz sampling rate from nine subjects. Data were recorded from each subject in two sessions; each session has 72 trials per class resulting in 288 samples per session. The timing scheme consists of a fixation of 2 s, a cue time of 1.25 s, followed by a period of a MI of 4 s.

It is confirming that all methods were carried out in accordance with relevant guidelines and regulations. Besides, all experimental protocols were approved by of the BCI competition IV-2a and it is confirmed that informed consent was obtained from all subjects and/or their legal guardian(s).

## Methods

In this section, the feature filtering preprocesses are mentioned at first, and then the temporal-spatial-frequency feature extraction and classification processing are explained in the form of proposed architecture.

### Multi-scale filter bank CSP

The organization of EEG signal logged through hand movement imagination was done by the Filter bank CSP technique^8^. Let ${X}^{c}=\left[{x}_{1}^{c},{x}_{2}^{c},\dots ,{x}_{n}^{c}\right]$ be EEG data matrices of an experiment where $\mathrm{C}=\mathrm{1,2},\dots ,\mathrm{C}$ is a number of classes, ${x}_{i}^{c}\in {R}^{D\times S}$ is a $D\times T$ matrix comprising information of ${i}_{t}h$ trial for class c where *D* represents the number of channels, and *T* stands for the number of time sample in each trials measurement. Averaged normalized covariance matrix belonging to class c can be shown as:

1 $$\overline{R}^{c} = \frac{{\mathop \sum \nolimits_{i = 1}^{N} \frac{{x_{i}^{c} x_{1}^{{c^{T} }} }}{{tr\left( {x_{i}^{c} x_{1}^{{c^{T} }} } \right)}}}}{N}$$

The subsequent optimization issue is solved to obtain *w*_*csp*_:

2 $$w_{csp} = \arg \mathop {\max }\limits_{w} \frac{{w^{T} R_{j} w}}{{w^{T} R_{i} w}}$$

**Figure 2:**
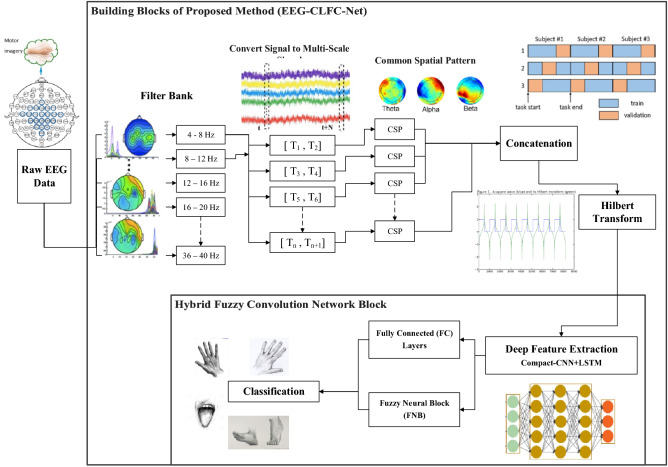
Building blocks of proposed work, Deep Frequency Band Convolutional Neural Network.

There are numerous techniques for resolving the maximization problem. Solving the following eigenvalue decomposition is a conventional way to find optimal $w_{csp}$ :

3 $$R_{i} W = \left( {R_{i} + R_{j} } \right)WD$$

where D is a generalized eigenvalue of *C*_*i*_. Those filters with higher Eigenvalues deliver greater alteration and vice versa as the Eigen vector at both ends offers discriminative features on one class against another. Communal training in the organization of motor imagery EEG signals is to chosen numerous Eigenvectors from both ends as a spatial filter. Besides, variable *M* signifies the number of choosen filters from both ends. Consequently, the spatially filtered signal Y in CSP subspace from a single EEG trial ${x}_{i}^{c}$ in sensor-space can be derived as:

4 $$Y = \omega \times x_{i}^{c}$$

where $\omega$ is the designated filter from *W*. Filter bank CSP (FBCSP) is an extension algorithm of CSP that was formerly presented by the winner of BCI competition IV-2a^9^. In our method, the used feature extraction process is primarily relies on FBCSP, and it will be clarified in the subsequent segment. As neural activities of different individuals are not identical in response and preparation time, selecting all sampled data in a trial to obtain our relevant signal is not essential. Therefore, FBCSP is extended to Multi-scale FBCSP (MSFBCSP) to utilize these neural activity phenomena^30^. Figure 2 demonstrates the Multi-scale FBCSP algorithm Block and other building blocks of our planned method mentioned in detail in the next section.

The procedure for MSFBCSP is as follows: The Matrix data of the EEG signals is ${N}_{Trial}\times T\times {N}_{CH}$ where ${N}_{Trial}$ is the number of trials recorded from subjects and *T* and ${N}_{CH}$ signify the number of time samples and recorded channels, respectively. Furthermore, ${N}_{F}$ represents the number of bandpass filters, usually recognized as Frequency Filter Bank. Typically, nine frequency filters with a bandwidth of 4_*HZ*_ like 4-8_*HZ*_, 8-12_*HZ*_,…,36-40_*HZ*_, are employed. Then, the Matrix is reshaped into ${N}_{Trial}\times {N}_{F}\times T\times {N}_{CH}$. In individual frequency bands, Multi-Scaled signals were composed by dividing these signals into chunks of a shorter time step. Consequently, the shape of constructed Matrix is ${N}_{Trial}\times {N}_{F}\times {N}_{T}\times T\times {N}_{CH}$ where ${N}_{T}$ expresses a number of time steps.

1. The spatial filter’s weights ${W}_{i,j}$ for time step *i* and frequency band j will be computed by the CSP algorithm. Then *M* most extreme eigenvalue and their corresponding pair are selected from each *W*_*i*, *j*_ and for every frequency band and time step. Therefore, they are ${2\times M\times N}_{CH}$ spatial filters.
2. Calculated spatial filters are employed in each time step, and frequency band on the Matrix data and Multi-Scaled Filter Banked signals are made with the new shape of the matrix data of ${N}_{Trial}\times {N}_{F}\times {N}_{T}\times T\times M\times 2$.

Variance across time is used to extract conventional energy of signals and therefore, the data Matrix develops in the maximum shape of ${N}_{Trial}\times {N}_{F}\times {N}_{T}\times {N}_{CH}\times 2$. It will be employed as a classifier input data.

### Extracting temporal, spatial and frequency band information

Once MSFBCSP transforming is performed, spatial filter weights that have already been calculated transferred EEG signals from sensor space to CSP subspace. A feature selection procedure is used for scaling down the search space after projecting the signal to the CSP subspace. Although the feature space dimensionality will be reduced, information loss might happen in waste features. Besides, CNN architectures are well recognized for their capabilities to massively decrease the number of factors in a model. Therefore, with using this kind of architecture, it is not vital to train a large number of factors. We elude information loss produced by feature selection algorithms and anticipate our network to consider weighing the features in the process.

Execution of the Hilbert transform on the aforementioned signal would extract in time dimension results in the envelope of each spatially filtered signal^9^. Performing Hilbert transform produces the analytic formula of the signal in complex-value, which simplifies envelope extraction by taking the amplitude of that complex-valued signal.

Down-sampling could be done on the signal without any important loss of information since the spectral nature of envelopes is low in frequency. There are two main advantages in this operation:

1. Down-sampling will combine the length of each signal as sampling duration varies between time steps.
2. our input feature dimension is reduced and therefore, the filters shape and the trainable parameter of the proposed model will be decreased.

The identical value of *N* = 2 and time-step intervals of 2.5–3.5 s, 3–4 s, 4–5 s, 5–6 s, 2.5–4.5 s, 4–6 s, 2.5–6 s after creation of imagery task has been adopted for this part^9^. The novel sampling rate of the data is 250 HZ, and it relies on the time step’s interval length whereas it will has dissimilar interval lengths. In addition, the cut-off frequency of the envelope is 4 HZ which means the least sampling frequency it needs to represent signals is 8 HZ (Nyquist Rate). However, different sampling frequencies are chosen for each time-step interval to get a united input Matrix. For example, for 4 s interval, a 10 Hz sampling rate is chosen to get 40-time points. Different sampling rates are chosen for other intervals to get unified time points.

### Compact convolution neural network

**Figure 3:**
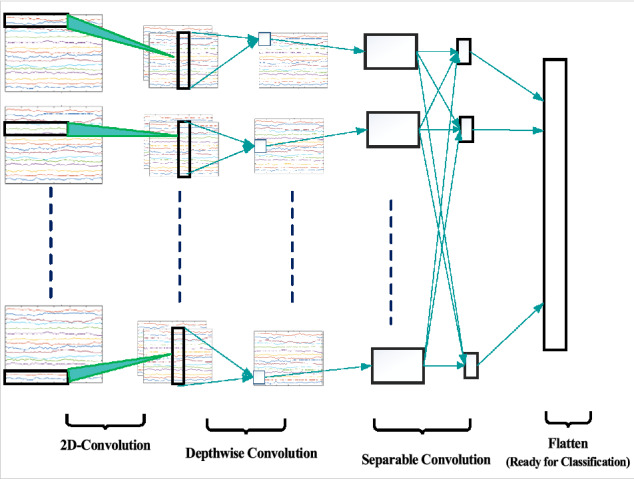
Overall visualization of the Compact-CNN architecture.

CNN is classified as a kind of artificial neural network, and it has a multilayer perceptron structure. This method is inherently enthused by the working standard of the visual cortex, and the convolutional layers are introduced into CNN. Weight-sharing and sparse connectivity are the advantages of the convolutional layers. The two benefits can meaningfully decrease computational difficulty. Dissimilar images and videos have a lot of data to train CNN, the amount of data on EEG signals is very small. For categorizing the EEG signals, several convolutional layers of CNN can easily result in over-fitting of the training model. Consequently, it is very significant to construct a suitable CNN model. Compact-CNN is a special CNN with depthwise and separable convolutions, and it has fewer parameters^16^. Figure 3 illustrates the example assembly of Compact-CNN on the BCI competition IV-2a data set.

As it is mentioned in Figure 3, the first part is inspired by Block 1 of EEGNet^16^. The order of two convolutional layers reproduces the models of the MSFBCSP. The initial convolutional layer achieves a convolution exclusively along the time axis. As an example, the explanation of the architecture of Compact-CNN on the BCI competition IV-2a data set is shown in Table 1. It is a regular convolutional layer with *F*_*1 *_= 8 kernels of size (1, 64) with padding that yields the same size as the input. Meanwhile, this strategy will permit the kernel to perform as a temporal filter that mines the related frequencies of the EEG signals. Then, the second convolutional layer makes a convolution along the electrodes (channels, space) axis only. A depthwise convolution was used with kernels of size (*C *= 22, 1) with no padding. In fact, this lets the CNN to learn numerous spatial filters by each feature map of the temporal convolution. In this block, *C *= 22 signifies the number of channels, *T *= 288 Number of trails, and the depth is *D *= 2 which means that there is an expansion of the number of feature maps from *F*_*1 *_= 8 to *D* * *F*_*1 *_= 16. This indicates that the layer will perform a linear grouping of the channels over the time where each channel will have its weight^16^. In the present study, three values are chosen via Coordinate-descent and Bayesian optimization: number of temporal filters, depth multiplier (number of spatial filters) and number of pointwise filters. The number of units in the output of the model is K = 4, and it is equal to the number of classes.

**Table 1:** The example architecture of Compact-CNN as a classifier on BCI competition IV-2a data set.

Layer	#Filters	input	Size	#Parameters	Output	Activation
Reshape		*C* × *T* = 22 × 288			1 × 22 × 288	
Conv2D	*F*_*1*_ = 8	1 × 22 × 288	(1,64)	512	8 × 22 × 288	Linear
BatchNorm		8 × 22 × 288		16	8 × 22 × 288	
DepthwiseConv2D	*D* * *F*_*1*=_16	8 × 22 × 288	(22,1)	352	16 × 1 × 288	Linear
BatchNorm		16 × 1 × 288		32	16 × 1 × 288	
Activation		16 × 1 × 288			16 × 1 × 288	ELU
AveragePool2D		16 × 1 × 288	(1,4)		16 × 1 × 72	
Dropout		16 × 1 × 72			16 × 1 × 72	
SeparableConv2D	*F*_*2*_ = 8	16 × 1 × 72	(1,16)	384	8 × 1 × 72	Linear
BatchNorm		8 × 1 × 72		16	8 × 1 × 72	
Activation		8 × 1 × 72			8 × 1 × 72	ELU
AveragePool2D		8 × 1 × 72	(1,8)		8 × 1 × 9	
Dropout		8 × 1 × 9			8 × 1 × 9	
Flatten		8 × 1 × 9			72	
Dense		72			4	Softmax

#### Parameter selection and training configuration

Tuning of numerous hyperparameters for neural networks and deep learning methods requires careful considerations. Unfortunately, this tuning is often a cumbersome task requiring expert experience, rules of thumb, or sometimes brute-force search. Consequently, inclination for automatic tactics with abilities to improve the enactment of any given learning algorithm has been arisen. An example of such a model is Multi-layer convolutional neural networks in which a comprehensive exploration of hyperparameters and architectures is useful, as it has been publicized with Bashashati et al^42^. For presented network architecture, a few features i.e. network hyperparameter such as type of pooling layer, number of the filter unit, convolution stride, kernel size, etc. should be chosen. It is approximately not possible and rational to find over the whole parameters to attain optimal values for each parameter. To resolve this issue, Bayesian parameter selection and Coordinate-descent are used as a suboptimal method to search through parameter space.

##### Parameter selection based on coordinate-descent

Hyperparameters, could be appropriately selected via cross-validation approach^9^. It is not possible to search over the factor space owing to time and computation limits. As an alternative, Coordinate-descent is used as a suboptimal technique to achieve cross validation for the network factors^43^. In this method, a set of parameters, $\theta = \left[ {\theta_{1} , \theta_{2} , . . . ,\theta_{N} } \right],$ is initialized and then the objective function or score function is optimized for each $\theta_{i} , \left( {i = 1, . . . , N} \right)$ independently, updating the values of the initial $\theta$ with the newly optimized parameters. After *N* optimizations, the $\theta$ vector will be completely updated and a new iteration of optimization can be initiated. To improve our results, the algorithm can be reiterated for several iterations^9^. In this work, three values are chosen via Coordinate-descent: number of temporal filters, depth multiplier (number of spatial filters) and the number of pointwise filters. Ten-fold cross validation is performed only once to opt the factors. The convolutional layer parameters are initial chosen via cross-validation and then the selected values are applied for cross validation to select the number of convolutional nodes.

##### Parameter selection based on bayesian optimization

In Bayesian method, unlike both random and grid explorations, preceding efforts are applied to reach optimal values in parameter form and space. It uses a probabilistic model for mapping hyperparameters to a probability of score on the objective function^42^. It is a capable algorithm that is skilled to optimize tasks and functions that are costly to evaluation via computational method and do not have identical structure to mathematical terms. In fact, this algorithm is extensively applied for optimization of hyper-parameters in the technique of machine learning. Its ability to comprise previous data about the optimization task aid this method to reserve its effectiveness even in a high number of functions. When the hyperparameters are optimized, the nominee points in the area of a definite point x have closely similar function values (namely the optimized function is smooth). In this method, a Kernel function is applied to join this domain information about the system. To gain a fresh candidate point, all the information attained from previous function approximations is applied in the Bayesian optimization. Indeed, the global knowledge reached in the likelihood distribution is applied to fit over the obtainable data to offer a fresh candidate^42^.

Unlike to the conventional optimization task, finding the maximum of the acquisition function is not difficult; however, this function is not still convex. For the optimization of this function, a derivative free optimization algorithm or a gradient descent algorithm could be used. The fresh nominee is a local maximum of the acquisition function. Then, the whole process is also done for the *T* iterations. Here, Bayesian optimization, with an optimization function of 10–tenfold cross-validation, has been performed to opt the best hyperparameters on the validation set. However, for regularization purposes and to avoid overfitting problems, batch normalization and dropout with a 0.5 rate have been employed after each convolutional layer.

### LSTM base CNNs

As a kind of regular neural network, the LSTM layer could learn long-term dependencies within the input data^24^. LSTM is an intermittent neural network with the capability to preserve the structure of data for a long time and classify the preferred pattern When the LSTM layer is loaded to a CNN design, the temporal features of the brain signals are professionally mined. At the center of the LSTM is the cell state which can be adapted by adding or eliminating information from the cell state. The removal or addition of the information from the cell states is normalized using structures named gates. The LSTM networks rely on stacked blocks containing three gates which are called the input gate, output gate, and forget gate. The aforementioned control cells are described by the following equations:

5 $$i = \sigma \left( {W_{i} x_{t} + U_{i} h_{t - 1} + b_{i} } \right)$$

6 $$f = \sigma \left( {W_{f} x_{t} + U_{f} h_{t - 1} + b_{f} } \right)$$

7 $$o = \sigma \left( {W_{o} x_{t} + U_{o} h_{t - 1} + b_{o} } \right)$$

8 $$\tilde{c} = W_{c} x_{t} + U_{i} h_{t - 1} + b_{c}$$

9 $$c_{t} = f \odot c_{t - 1} + i \odot \tilde{c}$$

10 $$h_{t} = o \odot c_{t}$$

11 $$\sigma \left( x \right) = \frac{1}{{1 + e^{ - x} }}$$

where $W$, $U$ and $b$ represent sets of learnable parameters to control each gate. $x, h, i, f, o$ and $c$ represents input, output, input gate, forget gate, output gate and memory cell state, respectively. $\odot$ represents element-wise product.$t$ represents the data as the time series^24^.

### Fuzzy neural block

A formal description of our planned FNB is explained in detail in this section. A fuzzy neural block (FNB) is known as an order of processing layers creating the activation of the antecedents of a fuzzy rule. Initially, the regularized output of the previous layer ***O*** is taken and then they are flattened as ${v}_{i}=Vec\left({O}_{i}\right)=[{o}_{\mathrm{1,1}},\dots ,{o}_{m,1},{o}_{\mathrm{1,2}},\dots ,{o}_{m,2},{o}_{1,n},\dots ,{o}_{m,n},]$. Next, the fuzzy clustering method by Kilic et al. is employed to obtain a set of K centroids of shape ${c}^{k}=[{c}_{1}^{k},{c}_{2}^{k},\dots ,{c}_{d}^{k}]$, via a collection *D* of ${v}_{i}$ of layer outputs obtained from the preceding period^29^. For the initial period, the centroids are set to zero. The Gaussian membership value of ${v}_{i}$ is computed as:

12 $$\mu_{i}^{k} \left( {v_{i} ,c^{k} ,\alpha } \right) = {\text{exp}}\left( { - 1/4\left( {v_{i} - c^{k} } \right)^{2} /\alpha^{2} } \right)$$

The scaling vector $\alpha$ is a parameter that is set to learn by the network, and the rule activation consists of a t-norm operator and normalization step such:

13 $$o^{k} \left( {\mathop \prod \limits_{j = 1}^{d} \mu_{i,j}^{k} } \right)\;{\text{and}}\;\tilde{o}^{k} = o^{k} /\mathop \sum \limits_{f = 1}^{k} o^{f}$$

where *d* is the dimension of the ${\mu }_{i}^{k}$ vector, and ${O}^{^{\prime}}=[{\widetilde{o}}^{1},\dots ,{\widetilde{o}}^{k}]$ is the output of the FNB that is forwarded to next layer. Note the output dimension of the FNB is reduced to $K$^25^.

### Proposed fuzzy convolution recurrent neural network (EEG-CLFCNet model)

**Figure 4:**
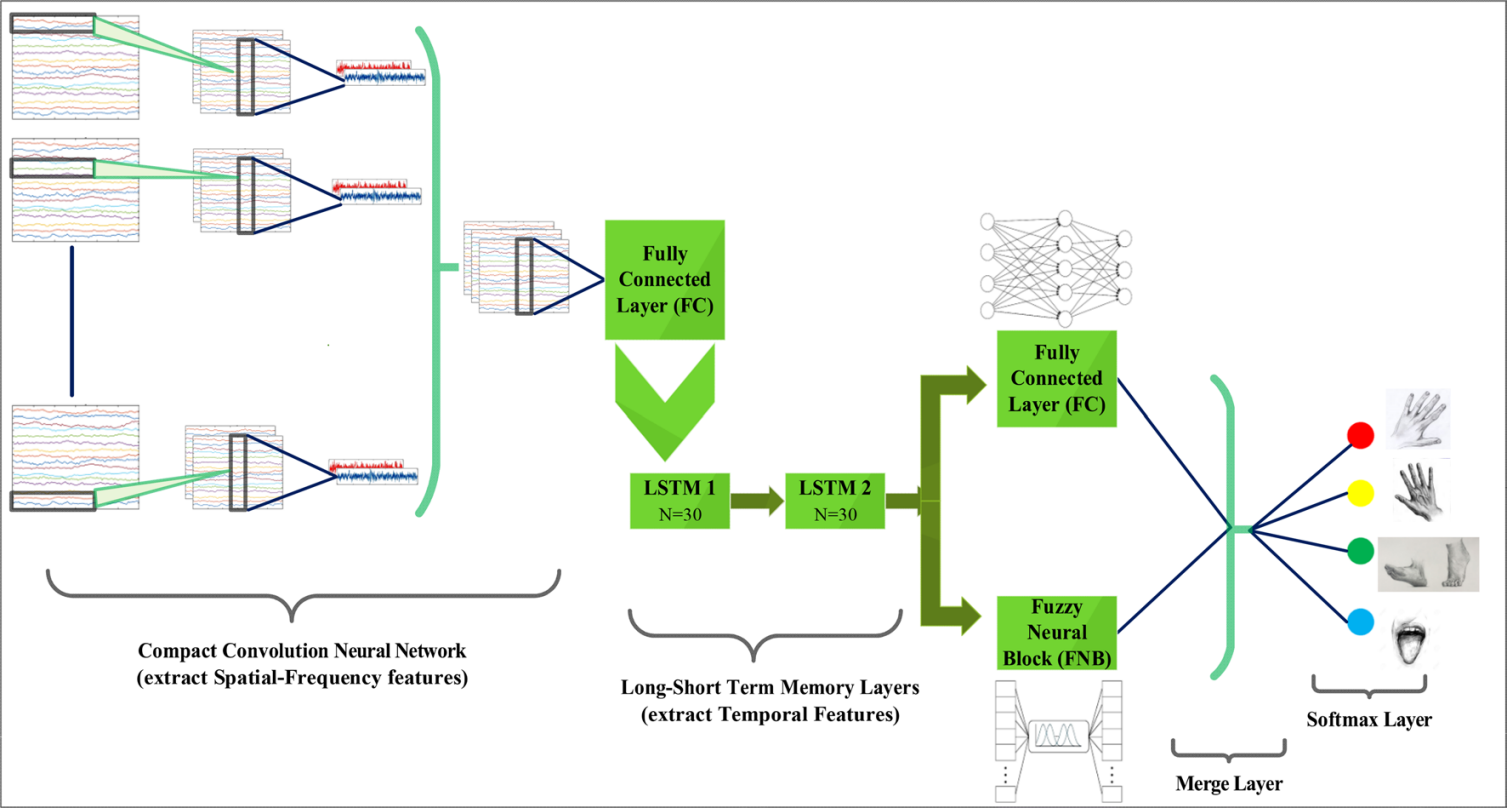
Visualization of Proposed Deep Fuzzy Convolutional Neural Network architecture.

The valuable information of EEG signals could be completely used afterward mining the temporal-spatial-frequency features. The key aim of our study is to develop grouping accuracy with complete feature extraction. Thus, how to join these three features is crucial. To attain the study goal, series convolutional recurrent neural network framework is compared and designed. The structure is shown in Fig. 4.

The spatial and frequency features of the filtered EEG signals are primarily extracted by Compact-CNN, and then the sequences of the extracted features is used as input in LSTM to extract temporal features. The output of the last time step of LSTM layers is transported to a fully connected layer and FNB. A softmax classifier finds the final prediction at last. Compact-CNN are used as the CNN module to define the series convolutional recurrent neural network with LSTM. The proposed method is named as EEG-CLFCNet, respectively.

## Results and analysis

In this section, the results analysis are explained in two cases: 1- to further interpret the experimental results over the brain regions with two proposed tuning approaches in terms of accuracy, some experiments are conducted, and 2- the results are compare to the state-of-the art methods to verify the proposed method validation.

### Experimental results

**Figure 5:**
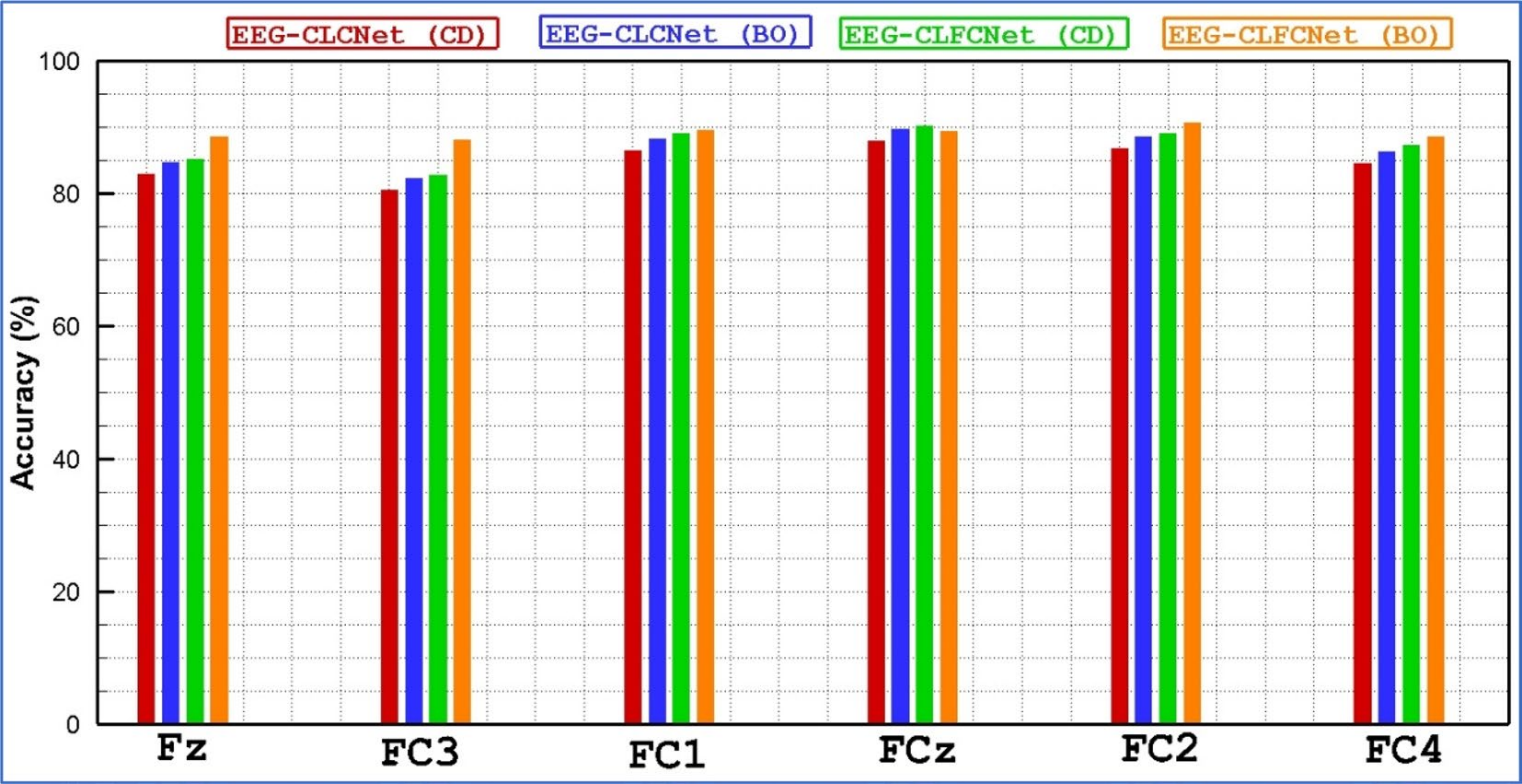
Accuracy of BCI Competition IV dataset 2a channels for the customized networks, with or without use of FNB and with two hyperparameter tuning methods. The horizontal axes presents the Frontal lobe channels (Fz, FC3, FC1, FCz, FC2, FC4) and the vertical axes presents the accuracy.

**Figure 6:**
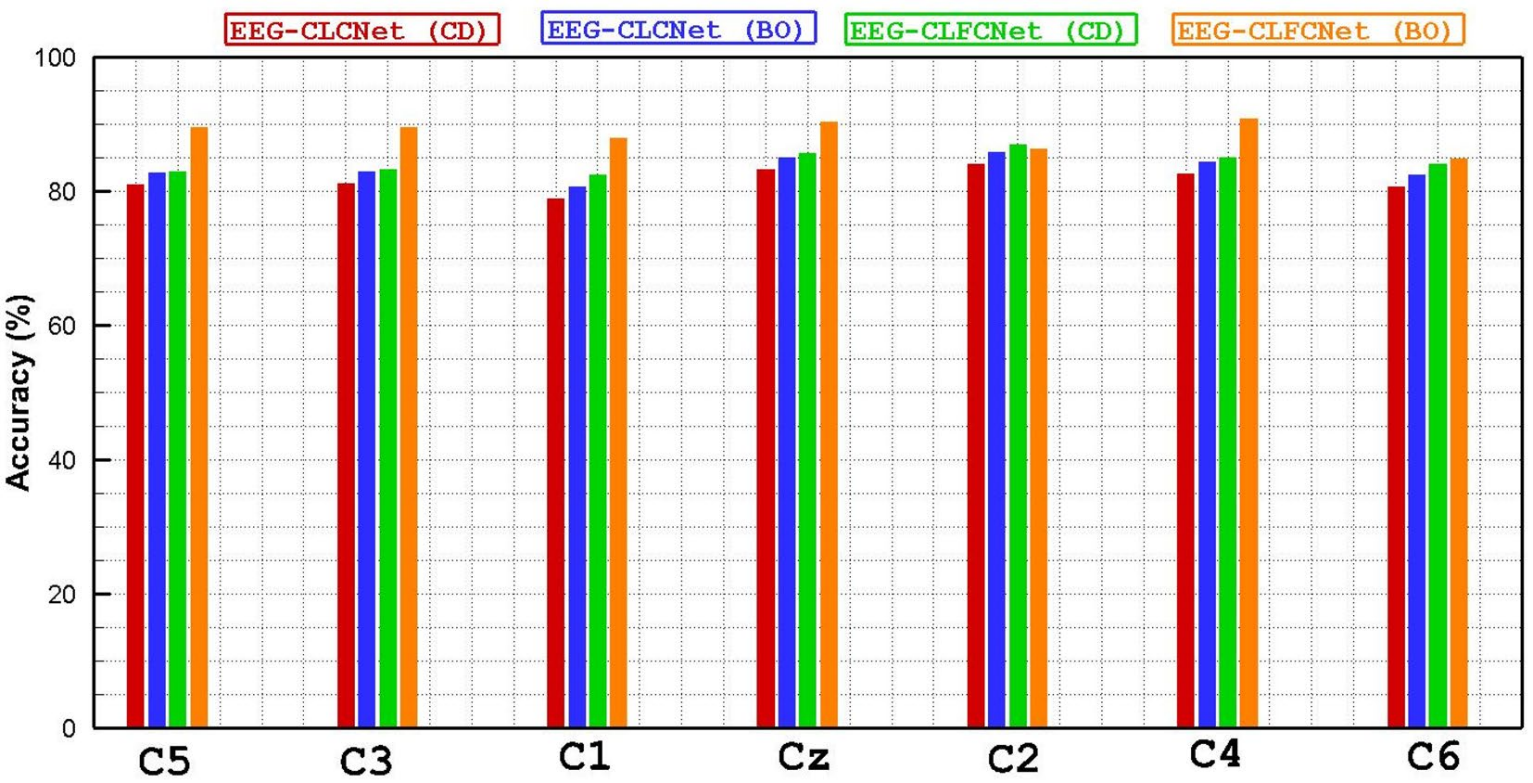
Accuracy of BCI Competition IV dataset 2a channels for the customized networks, with or without use of FNB and with two hyperparameter tuning methods. The horizontal axes presents the Frontal lobe channels (C5, C3, C1, Cz, C2, C4, C6) and the vertical axes presents the accuracy.

**Figure 7:**
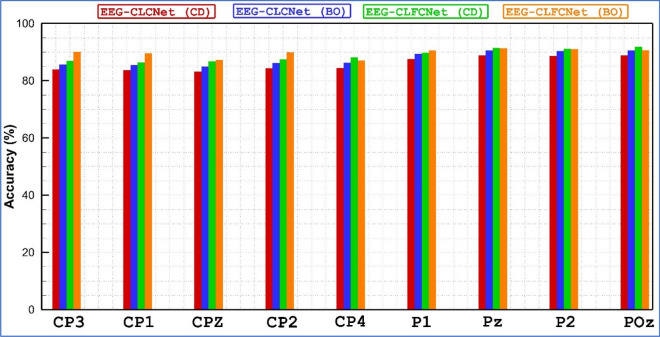
Accuracy of BCI Competition IV dataset 2a channels for the customized networks, with or without use of FNB and with two hyperparameter tuning methods. The horizontal axes presents the Frontal lobe channels (CP3, CP1, CPz, CP2, CP4, P1, Pz, P2, POz) and the vertical axes presents the accuracy.

To rationalize the result, a hybrid neural network with FNB including Compact-CNN and LSTM blocks has been trained and designed. Tables 2 and 3 the achieved results for the nine subjects (S01, S02, S03, S04, S05, S06, S07, S08, S09) and the twenty-two channels (Fz, FC3, FC1, FCz, FC2, FC4, C5, C3, C1, Cz, C2, C4, C6, CP3, CP1, CPz, CP2, CP4, P1, Pz, P2, POz) are shown in terms of accuracy for each EEG channel for considering the hyper-parameters tuning method, Coordinate-descent and Bayesian optimization, respectively. The presented results indicate that the channel variations of hybrid neural networks for both approaches are not high. In contrast, a hybrid neural network with BO attained better results compared to the CD. In addition, BCI Competition IV dataset 2a was recorded from 22 channels from the frontal lobe, central lobe, and parietal lobe. To examine the rank of each of these channels in the classification of motor imagery tasks, the accuracy for 22 EEG channels using customized methods has been calculated and displayed in Figs. 5, 6 and 7. A comparison of all EEG channels shows that FC1, FC2, FCz, P1, P2, Pz and POz attained greater accuracy (above 90%) by means of EEG-CLFCNet(CD). Moreover, channels of FC2, FCz, Cz, C4, CP1, CP2, CP3, P1, P2, Pz and POz attained higher accuracy (above 90%) when EEG-CLFCNet (BO) is employed.

**Table 2:** Accuracy of customized **EEG-CLFCNet (CD)** on each electrode for 9 subjects on BCI Competition IV dataset 2a.

Channels	Subj1	Subj2	Subj3	Subj4	Subj5	Subj6	Subj7	Subj8	Subj9	Mean on Subjs
Fz	92	85	88	86	90	83	82	76	94	86.2
FC3	95	84	80	81	91	73	78	80	97	84.3
FC1	97	85	94	89	94	84	89	80	97	**90**
FCz	96	87	89	87	93	95	91	84	98	**91.1**
FC2	91	86	93	84	95	92	89	83	96	**90**
FC4	90	91	90	80	89	89	88	81	93	87.8
C5	93	83	88	77	86	79	87	70	93	84
C3	91	87	88	75	86	75	85	79	91	84.1
C1	90	87	87	76	80	86	83	70	88.5	83
Cz	90	89	80	81	85	87	79	95	93	86.5
C2	95	89	87	81	84	84	81	95	88	87.1
C4	95	85	82	84	85	81	79	89	95	86.1
C6	84	86	82	81	80	85	80	94	91	84.7
CP3	89	88	84	82	85	84	89	94	89	87.1
CP1	91	89	89	78	85	85	87	87	93	87.1
CPz	92	85	87	79	84	85	89	93	92	87.3
CP2	93	86	88	84	89	81	88	90	87	87.3
CP4	95	92	86	84	86	82	88	92	87	88
P1	91	89	96	90	93	83	87	89	98	**90.7**
Pz	93	92	94	91	88	88	94	91	97	**90.8**
P2	93	89	93	95	86	89	90	94	95	**91.6**
POz	92	93.5	90	87	95	93	93	91	98	**92.5**
Mean on Channels	91.3	87.7	88.1	83	88	84.6	86.15	86	93.2	87.65

Significant values are in [bold].

**Table 3:** Accuracy of customized **EEG-CLFCNet (BO)** on each electrode for 9 subjects on BCI Competition IV dataset 2a.

Channels	Subj1	Subj2	Subj3	Subj4	Subj5	Subj6	Subj7	Subj8	Subj9	Mean on Subj
Fz	83	92	94	91	91	83	88	87	91	88.8
FC3	88	95	80	79	91	90	89	93	87	88
FC1	82	90	91	90	90	90	91	92	88	89.3
FCz	92	91	89	92	83	88	88	92	92	**90**
FC2	89	93	94	92	85	93	91	88	90	**90.5**
FC4	93	87	87	81	89	88	92	92	91	88.8
C5	91	83	91	93	90	88	89	91	92	89.7
C3	89	87	90	86	92	89	92	92	88	89.4
C1	85	85	91	92	86	87	89	88	86	87.6
Cz	90	89	91	90	92	92	90	89	93	**90.6**
C2	68	91	90	79	95	90	90	87	93	87
C4	88	92	93	93	91	88	89	93	92	**91**
C6	87	70	91	88	80	87	90	86	88	85.2
CP3	87	87	92	90	90	93	89	92	89	**90**
CP1	91	90	90	90	91	88	88	88	91	**90**
CPz	88	93	92	77	92	85	89	82	92	87.7
CP2	93	90	95	89	90	83	90	89	93	**90.2**
CP4	88	89	90	82	91	78	88	90	89	87.2
P1	88	94	95	88	92	90	88	89	89	**90.3**
Pz	87	93	96	93	95	89	86	91	89	**91**
P2	91	92	90	93	89	92	89	92	92	**91.1**
POz	92	90	91	85	94	94	89	90	94	**91**
Mean on channel	88	89	91	88	90	88	89	90	90	89.2

Significant values are in [bold].

The involvement of frontal, central, and parietal regions in the classification of motor imagery tasks should be determined. Hence, Table 4 signifies the average accuracy for an input image of these three stated brain regions via the three hybrid neural networks. Former investigations explained that the main motor cortex offers the greatest significant signal for the creation of skilled movements. This specific area receives input from all cortical areas that contributed to motor control, comprising the parietal, premotor, and frontal areas. Hence, output from the primary motor cortex affects the motor neurons in the spinal cord^44^. Therefore, an approximately equal role is noticed in the perception of motor tasks for the three mentioned lobes. However, the proposed EEG-CLFCNet (BO) attained the higher accuracy for all three brain regions with the highest average accuracy of 93% for the central lobe.

**Table 4:** Average accuracy of three brain regions with proposed methods.

Proposed methods	Proposed methods	Frontal lobe	Central lobe	Parietal lobe
With FNB	**EEG-CLFCNet (CD)**	85	87	91
	**EEG-CLFCNet (BO)**	86	91	92
Without FNB	**EEG-CLCNet (CD)**	84	84	85
	**EEG-CLFCNet (BO)**	83	86	88

### Comparison models

Several studies have been performed to progress the accuracy of classification in motor imagery tasks. Table 5 summarizes the specifics of these studies. As can be seen, these studies have used temporal or spatial info of EEG signals. Besides, EEG signals comprise numerous frequency bands, and each of these bands has exact biological significance. Time-spatial-frequency representation is the finest method to advantage of these frequency features accompanied by the spatial and temporal information of EEG signals. Moreover, the CNN learning process is facilitated when a 2-dimensional representation of EEG signals, since these networks are the most influential networks to learn spatial patterns of input images and Fuzzy block could consider the uncertainty and improve the ecological validity of MI-based BCI.

**Table 5:** Contrast with the advanced methods and baseline methods. All the methods are relied on the BCI Competition IV-2a datasets. Values in parenthesis are kappa value and the nonparanthesis are accuracy values.

Work	Methods	Activation function	Hyperparameters tuning method	ACC (Kappa)
Sakhavi et al. (2018)^9^	FBCSP + SVM	ReLU	Coordinate-descent	71.18(0.616)
Sakhavi et al. (2018)^9^	FBCSP + CNN	ReLU	Coordinate-descent	74.46 (0.659)
Sorkhi et al. (2022)^20^	MSFBCSP + TFCNN	ReLU	Bayesian Optimization	75.51 (0.673)
Schirrmeister et al. (2017)^1^	Shallow CNN Deep CNN	ELU	Sampling strategies	73.7 (0.65) 70.9 (0.61)
Riyad et al., (2020)^15^	Inception + EEG-net	ELU	Cross Validation	74.7 (0.66)
Lu et. al (2019)^36^	1LAYER-CNN + LSTM	ReLU	Cross Validation	76.62 (0.68)
Zhang et.al (2019)^18^	Over-FBCSP + HDNN	ReLU	Constant based on prestudeies	85(0.80)
Zhang et.al (2021)^19^	Over-FBCSP + HDNN-TL	ReLU	Fine-tune with 0.001 learning rate	85(0.81)
Wang et. al (2020)^24^	SCCRNN	ELU	Constant based on prestudeies	73(0.64)
Proposed work without FNB	EEG-CLFCNet	ELU	CD BO	**87.65 (0.835)** **89.2 (0.856)**
Proposed work with FNB	EEG-CLCNet	ELU	CD BO	84.2 (0.792) 85.7 (0.81)

Significant values are in [bold].

To improve the optimization performance of current models, a proposed idea was inspired in which a hybrid neural network consisting of Compact-CNN and LSTM networks is employed to learn the sequential features of EEG signals accompanied by spatial features^24^. Tables 6 and 7 present that our proposed networks outperformed hybrid neural networks in comparison with previous investigations in terms of kappa values and accuracy.

**Table 6:** Comparison between our networks and previous networks in terms of accuracy values.

Subject	Without FNB						With FNB	
	FBCSP + CNN^9^	MSFBCSP + TFCNN^20^	Over-FBCSP + HDNN^18^	SCCRNN^24^	EEG-CLCNet (CD)	EEG-CLCNet (BO)	EEG-CLFCNet (CD)	EEG-CLFCNet (BO)
S1	87.5	85.4	94	82.75	87	91	91	88
S2	65.28	63.77	72	53.5	83	87	87	89
S3	90.28	84.98	89	81.25	84	85	88.1	91
S4	66.67	64.48	75	73.75	78	81	83	88
S5	62.5	61.25	85	65.5	82	88	88	90
S6	45.49	59.30	81	60.25	80	82	84.6	88
S7	89.58	86.64	89	82	84	81	86.15	89
S8	83.33	88.93	90	83.5	88	86	87	90
S9	79.51	84.84	93	77.5	92	93	93.2	90
Mean	74.46	75.51	85	73	**84.2**	**85.7**	**87.65**	**89.26**

Significant values are in [bold].

**Table 7:** Comparison between our networks and previous networks in terms of kappa values.

Subject	Without FNB						With FNB	
	FBCSP + CNN^9^	MSFBCSP + TFCNN^20^	Over-FBCSP + HDNN^18^	SCCRNN^24^	EEG-CLCNet (CD)	EEG-CLCNet (BO)	EEG-CLFCNet (CD)	EEG-CLFCNet (BO)
S1	0.833	0.805	0.87	0.77	0.836	0.88	0.884	0.84
S2	0.537	0.516	0.59	0.38	0.773	0.826	0.836	0.853
S3	0.870	0.799	0.90	0.75	0.786	0.8	0.841	0.88
S4	0.556	0.526	0.76	0.65	0.706	0.746	0.773	0.84
S5	0.500	0.483	0.82	0.54	0.76	0.84	0.84	0.866
S6	0.273	0.446	0.66	0.47	0.733	0.76	0.794	0.84
S7	0.861	0.821	0.95	0.76	0.786	0.764	0.815	0.853
S8	0.778	0.852	0.86	0.78	0.84	0.813	0.836	0.866
S9	0.727	0.797	0.89	0.7	0.893	0.906	0.909	0.866
Mean	0.659	0.673	0.80	0.64	**0.783**	**0.81**	**0.835**	**0.856**

Significant values are in [bold].

In present study, we profited from the insights behind the preceding works and optimized them to attain a better result. To do so, initially, the input in terms of the time–frequency representation is optimized to facilitate the learning process by MSFCSP and Hilbert transform. Next, a hybrid neural network consisting of the CNN and LSTM neural networks is developed to extract spatial features and disclose sequence features of the EEG signals. Third, the Fuzzy neural block in merging is merged with a fully linked network to reflect the uncertainty and recover the performance. In the current study, two hyperparameters are used for tuning methods, Coordinate-descent and Bayesian Optimization. The results related to this comparison are presented in Tables 5, 6 and 7. Eventually, a higher accuracy was obtained from the hybrid network with FNB and BO method.

The main advantages of the presented network in comparison with other methods are the low parameter size, and the architecture of Compact-CNN used in our models. As Ref^24^ estimated, the trained model of Compact-CNN has higher generalization enactment. This is mainly due to the different structures of shallow CNN and compact CNN. Taking our own dataset as an example, the numbers of trainable parameters are 67,213 and 1946 for shallow CNN and compact CNN, respectively. Not only training parameters is less than shallow CNN in presented model, but also the training speed of Compact-CNN is also faster. Furthermore, classification accuracy is increased in the proposed method since fuzzy neural networks could produce not only crisp values but also the fuzzy values; this indicates that fuzzy set contained more information and it could increase the influence of function estimation.

To evaluate our model, a one-sided Wilcoxin signed-rank test is also used to verify and disclose the prominence for decrease/ increase of the accuracy^45^. This test is extremely commended when the number of paired samples for contrast is properly non-Gaussian and small. Now, *p*-values attained by execution Wilcoxon signed rank test between results of dissimilar methods are presented Table 8. The results of Wilcoxon test show that the rise of mean accuracy is statistically noteworthy (*p* < 0.05) compared to all approaches excluding for Over-FBCSP + HDNN as the *p*-value indicates in the Table 8.

**Table 8:** Cross models Wilcoxon signed ranked test. Each row demonstrate level of significance for a model against different models.

Metric	FBCSP + CNN^9^	MSFBCSP + TFCNN^20^	Over-FBCSP + HDNN^18^	SCCRNN^24^	EEG-CLFCNet(BO)
FBCSP + CNN^9^	Non	0.0117	0.112	0.015	0.0312
MSFBCSP + TFCNN^20^	0. 0117	Non	0.008	0.021	0.0117
Over-FBCSP + HDNN^18^	0.112	0. 008	Non	0.483	0.68
SCCRNN^24^	0.015	0.021	0.483	Non	0.023
EEG-CLFCNet	0.0312	0. 0117	0.68	0.023	Non

## Conclusion

In this paper, efficient method for interpret a user’s brain signal, extracting spatial–temporal-frequency features concurrently and considering the uncertainty of EEG property is proposed. Here, Multi-scale filter bank CSP presents along with a novel classification model in which the Compact-CNN, LSTM and Fuzzy neural block methods are joined, since Compact-CNN is responsible for extracting spatial-frequency features and LSTM network is in charge with temporal features. They combined sequentially, and then Fully-connected network and Fuzzy neural block are employed for target response classification. Adding a fuzzy component to the deep learning architecture improves its performance since it helps to cope with a higher parametric uncertainty of the model. The achieved results represent that using this type of architecture significantly improves the model’s accuracy compared with similar architectures on the BCI Competition IV-2a dataset. As results show, an average accuracy of 89.2% is attained by the proposed model via the usage of Bayesian optimization as a tuning hyperparameters method. Hence, the feasibility of applying BCI in real settings and natural surroundings is also disclosed. The presented results confirm the potential of deep learning as an effective classifier for Motor Imagery EEG signals and disclose the great capability of convolutional neural networks in learning signal’s temporal morphology and dependencies between features. Besides, our investigations confirm that the performance of architecture’s design is improved when it takes advantage of the signal’s template, specific to a frequency band. Furthermore, adopting the EEG signal’s multi-scaled form as an input to the neural network model expressively surges the SNR ratio of the signal in case of facing Gaussian artifacts or noise. Besides, the study of the four brain lobes indicated that the central lobe was the most complicated one in the discrimination of the motor imagery tasks. There are some limitations like any other studies, the nature of deep learning approaches is the first limitation relates. Training time is the key disadvantage of deep learning, despite using a Compact-CNN and its lower parameters. Nevertheless, once the network is trained, the classification phase is running in lower computational time. Another limitation of this research is the small size of the Competition IV dataset 2a. In future work, it is recommended to evaluate the proposed model with transfer learning approach, and with different mental tasks, such as attention or working memory. In addition, another input data strategy, for instance, to deal with applications where a class imbalance is inevitable could be investigated.

## Data Availability

The datasets generated and/or analysed during the current study are available in the *BCI Competition IV* repository, [https://www.bbci.de/competition/iv/#datasets].
